# Cytosolic phospholipase A_2_ plays a crucial role in ROS/NO signaling during microglial activation through the lipoxygenase pathway

**DOI:** 10.1186/s12974-015-0419-0

**Published:** 2015-10-31

**Authors:** Dennis Y. Chuang, Agnes Simonyi, Paul T. Kotzbauer, Zezong Gu, Grace Y. Sun

**Affiliations:** Interdisciplinary Neuroscience Program, University of Missouri, Columbia, MO USA; Center for Translational Neuroscience, University of Missouri, Columbia, MO USA; Center for Botanical Interaction Studies, University of Missouri, Columbia, MO USA; Department of Biochemistry, University of Missouri, Columbia, MO USA; Department of Neurology, Washington University School of Medicine, St. Louis, MO USA; Department of Pathology and Anatomical Sciences, University of Missouri, Columbia, MO USA

**Keywords:** Microglia, cPLA_2_, Arachidonic acid, Lipoxygenase, ROS, NO

## Abstract

**Background:**

Oxidative stress and inflammation are important factors contributing to the pathophysiology of numerous neurological disorders, including Alzheimer’s disease, Parkinson’s disease, acute stroke, and infections of the brain. There is well-established evidence that proinflammatory cytokines and glutamate, as well as reactive oxygen species (ROS) and nitric oxide (NO), are produced upon microglia activation, and these are important factors contributing to inflammatory responses and cytotoxic damage to surrounding neurons and neighboring cells. Microglial cells express relatively high levels of cytosolic phospholipase A_2_ (cPLA_2_), an enzyme known to regulate membrane phospholipid homeostasis and release of arachidonic acid (AA) for synthesis of eicosanoids. The goal for this study is to elucidate the role of cPLA_2_IV in mediating the oxidative and inflammatory responses in microglial cells.

**Methods:**

Experiments involved primary microglia cells isolated from transgenic mice deficient in cPLA_2_α or iPLA_2_β, as well as murine immortalized BV-2 microglial cells. Inhibitors of cPLA_2_/iPLA_2_/cyclooxygenase (COX)/lipoxygenase (LOX) were used in BV-2 microglial cell line. siRNA transfection was employed to knockdown cPLA_2_ expression in BV-2 cells. Griess reaction protocol was used to determine NO concentration, and CM-H2DCF-DA was used to detect ROS production in primary microglia and BV-2 cells. WST-1 assay was used to assess cell viability. Western blotting was used to assess protein expression levels. Immunocytochemical staining for phalloidin against F-actin was used to demonstrate cell morphology.

**Results:**

In both primary and BV-2 microglial cells, stimulation with lipopolysaccharide (LPS) or interferon gamma (IFNγ) resulted in a time-dependent increase in phosphorylation of cPLA_2_ together with ERK1/2. In BV-2 cells, LPS- and IFNγ-induced ROS and NO production was inhibited by arachidonyl trifluoromethyl ketone (AACOCF3) and pyrrophenone as well as RNA interference, but not BEL, suggesting a link between cPLA_2_, and not iPLA_2_, on LPS/IFNγ-induced nitrosative and oxidative stress in microglial cells. Primary microglial cells isolated from cPLA_2_α-deficient mice generated significantly less NO and ROS as compared with the wild-type mice. Microglia isolated from iPLA_2_β-deficient mice did not show a decrease in LPS-induced NO and ROS production. LPS/IFNγ induced morphological changes in primary microglia, and these changes were mitigated by AACOCF3. Interestingly, despite that LPS and IFNγ induced an increase in phospho-cPLA_2_ and prostaglandin E2 (PGE2) release, LPS- and IFNγ-induced NO and ROS production were not altered by the COX-1/2 inhibitor but were suppressed by the LOX-12 and LOX-15 inhibitors instead.

**Conclusions:**

In summary, the results in this study demonstrated the role of cPLA_2_ in microglial activation with metabolic links to oxidative and inflammatory responses, and this was in part regulated by the AA metabolic pathways, namely the LOXs. Further studies with targeted inhibition of cPLA_2_/LOX in microglia during neuroinflammatory conditions can be valuable to investigate the therapeutic potential in ameliorating neurological disease pathology.

**Electronic supplementary material:**

The online version of this article (doi:10.1186/s12974-015-0419-0) contains supplementary material, which is available to authorized users.

## Background

Neuroinflammation plays a major role in the progression of neurodegenerative diseases including Alzheimer’s disease, Parkinson’s diseases, cerebral vascular stroke, and infectious HIV encephalopathy. Microglial cells, the resident innate immune cells in the central nervous system (CNS), are known to exert multiple physiologic functions in the brain, including anchoring CNS innate immune response through phagocytosis of foreign pathogens, removing cellular breakdown products, stimulating tissue repair process, and maintaining tissue homeostasis [1]. Activation of microglial cells can also exert significant impact on the propagation of inflammatory responses [2, 3]. For instance, activated microglia was shown in vivo to contribute to expansion of infarct after focal cerebral ischemia [4], and inhibition of microglial activation was proven a viable strategy to prevent inflammatory neuronal death in vitro [5]. Recent studies had place much focus on the differential functions of polarized M1/M2 microglial cells after activation. While much research is currently underway to distinguish the biochemical and functional properties of each phenotype, most tend to agree that M1 microglia are more cytotoxic and persist during the disease effector stage, whereas M2 microglia are more neuroprotective and predominate during the repair stage [6–8]. The discovery of functional differences and delineation of time course of microglia polarization has generated interest in ways to limit M1 activation and stimulate M2 transformation in order to ameliorate outcomes of neurological diseases, including experimental stroke and traumatic brain injury [9–13].

Biochemically, M1 microglial activation is associated with the release of ROS, NO, glutamate, cytokines (such as TNFα), phospholipases, matrix metalloproteases, and other proinflammatory factors contributing to the progressive neuronal damage observed in many neurodegenerative disorders [14–16]. Therefore, suppressing or limiting microglial activation can have beneficial effects for preventing neuroinflammation and neurodegeneration. Microglia in vitro can be activated with a variety of agents, such as proinflammatory cytokines (TNFα, IL-1β, IFNγ), lipopolysaccharides (LPS), and oligomeric beta amyloid (Aβ) [17]. Studies including those from our laboratory have demonstrated that microglia activation by proinflammatory cytokines and LPS causes induction of iNOS and activation of NADPH oxidase, leading to increased oxidative/nitrosative stress [18].

Phospholipase A_2_s (PLA_2_s) are groups of enzymes that hydrolyze the fatty acids from the *sn*-2 position of membrane phospholipids. Among the PLA_2_s identified, cPLA_2_ and iPLA_2_ are the constitutively active PLA_2_s that serve as important mediators for the release of polyunsaturated fatty acids, including arachidonic acid (AA) and docosahexaenoic acid from membrane phospholipids [19, 20]. Multiple studies have demonstrated group IV PLA_2_α (cPLA_2_α) to be the major PLA_2_ responsible for the release of AA and to play an essential role in inflammation. Transgenic mice lacking cPLA_2_α have been shown to display significantly reduced deleterious phenotypes in inflammatory diseases, such as ischemic brain injury, anaphylaxis, arthritis, alcoholism, and acute lung injury [21–27]. More recent in vivo studies demonstrated ability for pharmacological inhibitors of cPLA_2_ to ameliorate ischemic stroke, experimental autoimmune encephalitis, and spinal cord injury [28–30]. Although cPLA_2_ activation in the brain is associated with oxidative stress, neuronal excitation, and neuroinflammation [31], little is known about mechanism(s) for its activation in microglial cells [32]. Previous studies demonstrated protective effects of cPLA_2_ inhibition against microglia-induced white matter damage in vivo and oligodendrocyte cell death in vitro, suggesting the role of this enzyme as a potential target to suppress microglia-induced secondary damage in the central nervous system [30]. However, the mode of action of cPLA_2_ and its link to the inflammatory responses in microglial cells have not been elucidated in detail. In this study, we isolated primary microglial cells from cPLA_2_ and iPLA_2_ KO mice to demonstrate the role of cPLA_2_ (and not iPLA_2_) in mediating oxidative and inflammatory responses from LPS and IFNγ stimulation. In addition, we further suggested a mechanism that links cPLA_2_-mediated eicosanoid production with downstream ROS and NO generation in BV-2 microglial cells.

## Methods

### Materials

Dulbecco’s modified Eagle’s medium (DMEM), penicillin/streptomycin, and 0.25 % (*w*/*v*) trypsin/EDTA were obtained from GIBCO (Gaithersburg, MD). Endotoxin-free fetal bovine serum was from Atlanta Biologicals (Lawrenceville, GA). Lipopolysaccharide (LPS) (rough strains) from *Escherichia coli* F583 (Rd mutant) was purchased from Sigma-Aldrich (St. Louis, MO). Interferon-γ (IFNγ) was purchased from R&D Systems (Minneapolis, MN). Pharmacological inhibitors used include the following: U0126, SB202190, and SP600125 were from Cell Signaling (Beverly, MA). Arachidonyl trifluoromethyl ketone (AACOCF3), pyrrophenone, racemic bromoenol lactone (BEL), nordihydroguaiaretic acid (NDGA), ibuprofen, zileuton, and PD146176 were from Cayman Chemical (Ann Arbor, MI). NCTT-956 was from Sigma-Aldrich (St. Louis, MO). RNA interference Lipofectamine RNAiMAX Transfection Reagent was from Life Technology (Carlsbad, CA). siRNA against cPLA_2_ Mm_Pla2g4a_8 FlexiTube siRNA (NM_008869) and AllStars Negative Control siRNA were purchased from Qiagen (Hilden, Germany). Antibodies used for Western blots include the following: goat anti-rabbit IgG-horseradish peroxidase, goat anti-mouse IgG-horseradish peroxidase, anti-cPLA_2_ rabbit polyclonal, anti-iNOS rabbit polyclonal antibodies (Santa Cruz Biotechnology, Santa Cruz, CA); monoclonal anti-β-actin peroxidase (Sigma-Aldrich, St. Louis, MO); rabbit polyclonal anti-p-cPLA_2_, rabbit polyclonal anti-ERK1/2, and mouse monoclonal anti-phospho-ERK1/2 antibodies (Cell Signaling, Beverly, MA). An affinity-purified antibody directed against an iPLA_2_β peptide corresponding to residues 277–295 was a gift of Drs. Chris Jenkins and Richard Gross (Washington University School of Medicine, St. Louis, MO) [33]. For immunocytochemical staining, rabbit anti-ionized calcium-binding adapter molecule 1 (Iba-1) antibodies (019–19741) was purchased from Wako BioProducts (Richmond, VA), Alexa Fluor 488® phalloidin from Life Technologies (Carlsbad, CA), and 4′,6-diamidino-2-phenylindole (DAPI) from Roche Molecular Chemicals (Basel, Switzerland). For ROS detection, CM-H2DCF-DA (DCF) was purchased from Invitrogen, Inc. (Eugene, OR). WST-1 assay was purchased from Clontech (Mountain View, CA). Prostaglandin E2 (PGE2) EIA Kit was purchased from Cayman Chemicals (Ann Arbor, MI).

### cPLA_2_ transgenic animal breeding and genotyping

All animal care and experimental protocols were carried out in accordance with NIH guidelines and with permission from the University of Missouri Animal Care and Use Committee (protocol #6728). Pairs of C57Bl/6 male and female heterozygous cPLA_2_+/− mice were kindly provided by Dr Joseph V. Bonventre (Harvard Medical School, Boston, MA) and colony was expanded at the University of Missouri for more than five generations prior to start of the experiments. Wild-type cPLA_2_+/+ and homozygous knockout cPLA_2_−/− mice used in the experiments were generated by crossing male and female heterozygous cPLA_2_+/− mice, and genotyping of litters was done between postnatal day 3~6 by polymerase chain reaction (PCR) as previously described [21].

### iPLA_2_ transgenic animal breeding and genotyping

iPLA_2_β-KO mice were housed and cared for in animal facilities administered through the Washington University Division of Comparative Medicine, and animal procedures were performed according to a protocol approved by the Washington University Animal Studies Committee. iPLA_2_β-KO mice were previously generated by insertion of the neomycin resistance gene into exon 9 of the mouse iPLA_2_β (*Pla2g6*) gene by homologous recombination [34, 35]. KO and WT mice were generated by mating heterozygous mice, and their genotype was determined by a PCR assay. The primers used for PCR genotyping were WT F1 (TTACCTCCGCTTCTCGTCCCTCATGGAGCT), Neo F1 (GGGAACTTCCTGACTAGGGGAGGAGTAGAA), and WT R1 (TCTGTTTCTCTAGAGACCCATGGGGCCTTG), which when combined in a single PCR reaction generate a 158-bp band for the WT allele and a 254-bp band for the KO allele.

### Primary microglia isolation

Preparations of primary microglial cells with postnatal day 7–10 C57Bl/6 pups were accomplished with the Miltenyi Biotec MACS cell separation system (Bergisch Gladbach, Germany). Briefly, brains from the genotyped pups were dissected and meninges removed. Tissues were dissociated using the Neural Tissue Dissociation Kit (P) (Miltenyi Biotec) with the gentleMACS dissociator. Prior to isolation, cell concentration in the suspension was counted and roughly 10^5^ cells were collected for flow cytometry analysis by the Cellular Immunology Core in the University of Missouri. Microglia were isolated from the single-cell suspension using the magnetic activated cell sorting (MACS) technology with anti-cluster of differentiation molecule 11b (CD11b) (Microglia) MicroBeads (Miltenyi Biotec) in combination with an OctoMACS Separator with slight modifications to the manufacturer’s instructions. The number of cells post-isolation was counted, and roughly 10^5^ cells were collected for post-isolation flow cytometry analysis, and the remaining cells were plated at a density of 5 × 10^5^/cm^2^. Plated cells were cultured in DMEM supplemented with 10 % FBS containing 100 units/mL penicillin and 100 μg/mL streptomycin and maintained in 5 % CO_2_ incubator at 37 °C. Culture medium was replaced every 3–5 days. Primary cell cultures were used between days-in-vitro (DIV)5–7.

### Immortalized microglial BV-2 cell culture

The murine BV-2 cell line was generated by infecting primary microglia cell cultures with a v-raf/v-myc oncogene-carrying retrovirus (J2) [36]. These cells were obtained as a gift from Dr. R. Donato [37] and prepared as previously described [18, 38]. Briefly, cells were cultured in DMEM (high glucose) supplemented with 10 % FBS containing 100 units/mL penicillin and 100 μg/mL streptomycin and maintained in 5 % CO_2_ incubator at 37 °C. For subculture, cells were removed from the culture flask by gentle scraping, re-suspended in the culture medium, and sub-cultured in 6/96-well plates for experiments. Cell condition and morphology were assessed by using a phase contrast Nikon DIAPHOT 300 microscope attached with a CCD cool camera, and MagnaFire2.1C software was used for image capture and processing. Representative bright field pictures were obtained using a 20× objective lens.

### Flow cytometry for microglial cell purity analysis

During primary microglia isolation, after tissue dissociation and cell number were determined, roughly 10^5^ cells were collected, resuspended in 100 μL buffer, and incubated with 10 μL CD11b-fluorescein isothiocyanate (FITC) antibodies (Miltenyi) for 10 min in 4 °C. Cells were then washed and resuspended in 100 μL fresh buffer for flow cytometry analysis. Similarly, after microglia isolating by CD11b cell sorting, 10^5^ cells were collected and labeled with cd11b-FITC for flow cytometry analysis. Flow cytometry analysis was performed using the BD FACScan under the FITC protocol by the Cellular Immunology Core in the University of Missouri.

### cPLA_2_ RNA interference knockdown in BV-2 cells

BV-2 cells were seeded in 96- and 24-well plates with antibiotic-free DMEM containing 5 % FBS for 24 h. When cell density reached roughly 70–80 %, they were transfected with either AllStars negative control siRNA (Qiagen) or cPLA_2_ siRNA (NM_008869, Qiagen) (final concentration of 40 nM) using the RNAiMAX transfection reagent (Invitrogen) in mixture of Opti-MEM and DMEM mediums for 48 h prior to being used for experiments, according to the manufacturer’s instructions. cPLA_2_ knockdown was evaluated by Western blot for protein expression of total cPLA_2_ normalized against β-actin.

### Cell viability assay protocol

The WST-1 protocol was used for assessment of cell viability. Briefly, after reaching 80–90 % confluence, cells in 96-well plates were serum starved for 4 h, followed by incubation with inhibitors for 16 h. After treatment, cell viability was determined by adding 10 μL of the WST-1 reagent (Roche Applied Science, Germany) into each well. After gentle shaking, cells were incubated for 1 h at 37 °C and absorbance was read at 450 nm (with reference wavelength at 650 nm).

### NO determination

NO released from BV-2 cells was converted to nitrite in the culture medium. NO concentration was measured using the Griess reagent protocol as described previously [18]. In brief, BV-2 cells in 96-well plate were serum starved in phenol red-free DMEM for 3 h, followed by incubation with designated inhibitors for 1 h. Cell were then incubated with IFNγ or LPS at 37 °C for 16 h. Alternatively, primary microglia were stimulated with IFNγ or LPS at 37 °C for 24/48 h. Aliquots of medium (50 μL) were incubated with 50 μL of the reagent A [1 % (*w*/*v*) sulfanilamide in 5 % phosphoric acid, Sigma-Aldrich] for 10 min at room temperature covered in dark. This was followed by addition of 50 μL of reagent B [0.1 %, *w*/*v*, N-1-naphthylethylenediamine dihydrochloride, Sigma-Aldrich] for 10 min at room temperature, protected from light, and absorbance at 543 nm was measured using a microplate reader (Biotek Synergy 4, Winooski, VT). Serial dilutions of sodium nitrite (0–100 μM) were used to generate the nitrite standard curve.

### ROS determination

ROS production in microglial cells was assessed with 5-(and-6)-chloromethyl-2′,7′-dichlorodihydrofluorescein diacetate (CM-H2DCF-DA, or DCF in short). Primary microglia or BV-2 microglial cells were seeded in 96-well plate and grown until 90 % confluent. Cells were serum starved for 3 h, followed by pretreatment with inhibitors for 1 h, prior to stimulation with LPS or IFNγ for 11 h. Alternatively, primary microglia were stimulated with IFNγ or LPS at 37 °C for 24/48 h. DCF (10 μM) was added to each well and incubated for 1 h. The fluorescent intensity of DCF was measured with a microplate reader (excitation wavelength of 490 nm and emission wavelength of 520 nm). Fluorescent intensity was normalized against control wells for statistical analysis.

### PGE2 ELISA protocol

PGE2 concentration in the cell-conditioned medium was assessed with the PGE2 ELISA protocol (Cayman Chemicals). Briefly, 50 μL of conditioned medium from treated BV-2 cells in 96-well plates were incubated with 50 μL of PGE2 monoclonal antibody and 50 μL of PGE2 AChE tracer for 18 h at 4 °C with plate covered with plastic film. Standard curve was generated with serial dilution of PGE2 EIA standard (Cayman No. 414014) in EIA buffer prepared according to Cayman’s protocol. On day 2, 100 dtn of Ellman’s Reagent was reconstituted with 20 mL of UltraPure water and 200 μL added into each sample/standard well for development. The plate was placed on an orbital shaker for 60 min prior to measuring for absorbance at 410 nm using a microplate reader. Concentration was calculated from the fourth-degree polynomial curve generated from the standard wells in accordance to kit instruction.

### Western blot analysis

Cell lysates were collected in RIPA buffer containing 50 mM Tris–HCl (pH 7.5), 150 mM NaCl, 1 % Nonidet P-40, and 0.1 % SDS. The extract was centrifuged at 10,000×*g* for 15 min at 4 °C and transferred to a clean tube to remove cell debris. Protein concentration was measured and normalized with the BCA protein assay kit (Pierce Biotechnology, Rockford, IL). Depending on the target of interest, 5–10 μg of total protein was loaded in SDS-PAGE for electrophoresis. After electrophoresis, proteins were transferred to 0.45-μm nitrocellulose membranes. Membranes were incubated in Tris-buffered saline containing 0.1 % Tween 20 (TBS-T) and 5 % non-fat milk for 1 h at room temperature. The blots were incubated at 4 °C overnight with antibodies cPLA_2_ (1:1000), phospho-cPLA_2_ (1:1000), ERK1/2 (1:2000), phospho-ERK1/2 (1:1000), iNOS polyclonal (1:1000), and β-actin (1:50,000). After repeated washing with 1X TBS-T, blots were incubated with goat anti-rabbit IgG-HRP (1:4000) or goat anti-mouse IgG-HRP (1:2000) for 1 h at room temperature. Immunolabeling was detected by chemiluminescence ECL/WestPico/Femto and developed in X-ray film developer. Films were scanned, and the optical density of bands was measured with the QuantityOne software (BioRad, Hercules, CA).

### Immunocytochemistry staining

Immunocytochemistry staining was carried out as previously described by Chuang et al. [39]. Briefly, cells were cultured in 24-well plates containing round cover slips. After treatment, cells were fixed in 4 % paraformaldehyde for 15 min and then permeabilized with 0.1 % Triton X-100 in PBS for 30 min. Cells were incubated with 10 % normal goat serum in 0.005 % Triton X-100 in PBS for 60 min then incubated overnight in 0.5 % normal goat serum in 0.005 % Triton X-100 in PBS containing primary antibodies. The next day, cells were incubated in 0.005 % Triton X-100 in PBS containing secondary antibodies, goat-anti-rabbit Alexa fluor 488 (Jackson Immunoresearch), and goat-anti-mouse Alexa fluor 549 (Jackson Immunoresearch) for 60 min, followed by 1 unit of Alexa Fluor 488 phalloidin (Life Technologies)/well for 20 min (5 μL of 6.6 μM stock solution dissolved in methanol diluted in 200 μL of 0.005 % Triton X-100 in PBS), and nuclear counterstaining with 1 μg/mL of 4,6-diamidine-2-phenylindole dihydrochloride (DAPI) (Pierce) in PBS for 10 min. The coverslips were then mounted on fluoromount (Sigma-Aldrich) and sealed with nail polish. Fluorescence photomicrographs were captured using a Leica DMI 6000B fully automated epifluorescence microscope (Leica Microsystems Inc., Buffalo Grove, IL).

### Statistical analysis

Data were presented as means ± SEM. Results were analyzed either by one-way ANOVA followed by Dunnett’s multiple comparison tests (V4.00; GraphPad Prism Software Inc., San Diego, CA). Statistical significance was considered for *P* < 0.05.

## Results

### Stimulation of iNOS, p-ERK1/2, and p-cPLA_2_ protein expression by LPS and IFNγ in primary and immortalized (BV-2) microglial cells

**Fig. 1:**
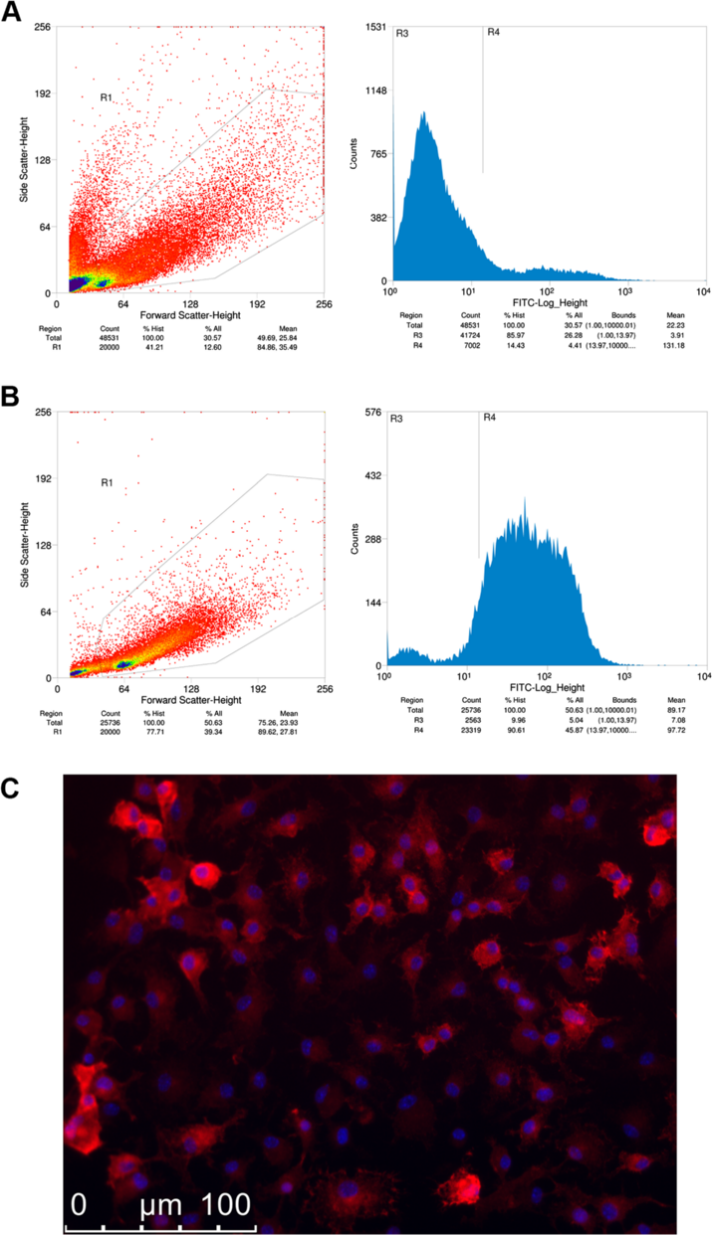
Determination of primary culture purity after isolation from brains of postnatal day 7–10 c57bl/6 mice. **a** Flow cytometry of cells from pre-sorting showed 14.43 % of cells in cell suspension positive for cd11b-FITC. **b** Flow cytometry of cells from post-sorting showed 90.61 % of cells in cell suspension positive for cd11b-FITC. **c** Immunocytochemical staining of cells in DIV5 primary culture showed expression of cd11b (*red*) in almost all cells

In this study, we first characterized primary microglial cells isolated from 7–10 days postnatal c57bl/6 WT and cPLA_2_ KO mouse brains using the Miltenyi Biotec MACS protocol. Flow cytometry analysis showed 14 % of cells from the homogenized brain tissues to express CD11b surface antigen (microglia) prior to cell sorting/isolation (Fig. 1a). After MACS isolation, more than 90 % of isolated cells were CD11b positive, indicating relatively high purity from the isolation protocol (Fig. 1b). Immunocytochemistry staining with antibodies targeted against CD11b followed by fluorescent microscopy demonstrated staining in nearly all cells with morphology that resembled ramified microglial cells (Fig. 1c).

**Fig. 2:**
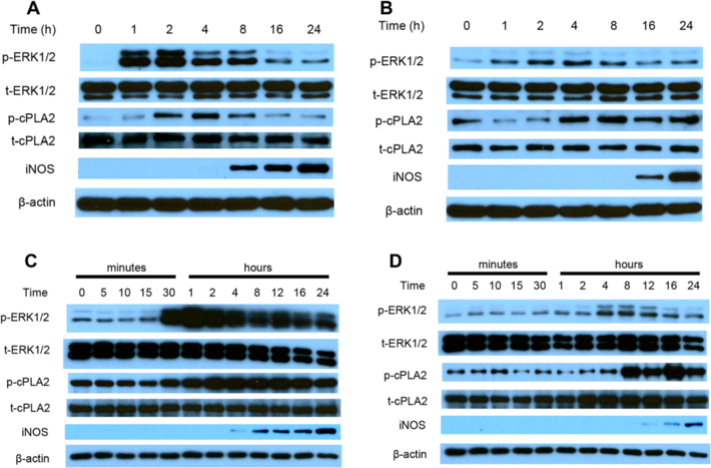
Time course of protein expression in primary microglia and BV-2 cells after LPS/IFNγ stimulation. Primary microglial cells were stimulated with **a** 200 ng/mL LPS or **b** 20 ng/mL IFNγ. Cells were lysed, and proteins were collected and processed at indicated time post-stimulation. Western blot was performed to determine protein expression. Similarly, the same procedure was performed with BV-2 cells stimulated with **c** 200 ng/mL LPS or **d** 20 ng/mL IFNγ. Results are representative blots of two independent time course experiments for primary microglia and three experiments for BV-2 cells

Our earlier studies have demonstrated the ability for BV-2 microglial cells to upregulate iNOS and produce NO after LPS or IFNγ stimulation individually and proposed ERK1/2 as one of the central components in mediating this transcriptional process [39, 40]. In this study, a time course study was carried out to test induction of iNOS, p-ERK1/2, and p-cPLA_2_ by LPS and IFNγ using primary cells and compare with BV-2 microglial cells. When primary microglial cells were stimulated with LPS (200 ng/mL), ERK1/2 was phosphorylated within an hour and started to decline after 2 h albeit remaining elevated up to 24 h compared with baseline (Fig. 2a). Following the increase in pERK1/2, LPS also induced increase in cPLA_2_ phosphorylation, with maximal expression occurring at 4 h prior to a gradual decline (Fig. 2a). On the other hand, iNOS expression was not observed until after 8 h and was highest at 24 h (Fig. 2a). A similar expression profile was observed when primary microglia was stimulated with IFNγ (20 ng/mL), although the sequence of events appeared to be delayed with ERK1/2 phosphorylation maxing at 4 h, cPLA_2_ phosphorylation maxing at 8 h, and iNOS expression not observed until 16 h post-stimulation (Fig. 2b). With IFNγ, p-cPLA_2_ remained upregulated at 24 h post-stimulation. There were no significant changes in total ERK1/2 or cPLA_2_ protein during the activation process. When the same conditions were subjected to BV-2 cells, matching trends were observed among the proteins of interest (Fig. 2c, d), with exception that LPS stimulation of p-ERK1/2 and p-cPLA_2_ appeared to be stronger than the primary microglial cells and remained upregulated at 24 h. These results suggest the use of BV-2 cells as a justified model system to investigate biochemical profiles of our pathway of interest during microglial activation.

### cPLA_2_ phosphorylation is regulated by phospho-ERK1/2

**Fig. 3:**
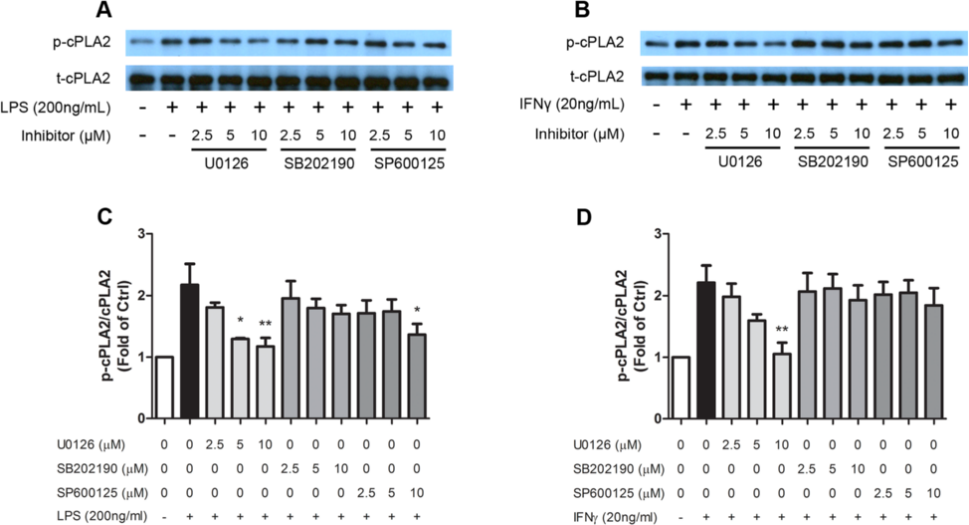
ERK1/2 contributed to cPLA_2_ phosphorylation after LPS/IFNγ stimulation. BV-2 cells were starved for 3 h in serum-free DMEM. One hour prior to stimulation, cells were pretreated with indicated concentrations of MAPK inhibitors: U0126 (U) for ERK1/2 inhibition, SB202190 (SB) for p38 MAPK inhibition, and SP600125 (SP) for JNK inhibition. Cells were then stimulated with **a**, **c** 200 ng/mL LPS or **b**, **d** 20 ng/mL IFNγ. Cells were lysed and proteins were collected and processed 2 h after LPS stimulation or 8 h after IFNγ stimulation for Western blot analyses. **a**, **b** Representative blots. Protein expression was quantified with QuantityOne software for three separate experiments for **c** LPS-stimulated BV-2 cells and **d** IFNγ-stimulated BV-2 cells. Results were expressed as the mean ± SEM (*n* = 3) and significant difference from the respective group was determined by one-way ANOVA followed by Dunnett’s post-tests, *P < 0.05; **P < 0.01

Among multiple sites, Ser505 residue is the site phosphorylated by MAPKs in cPLA_2_ [41]. In this study, we examined the effects of MAPK inhibitors, U0126 (MEK1/2-ERK1/2 inhibitor), SB202190 (p38 MAPK inhibitor), and SP600125 (JNK inhibitor) on cPLA_2_ phosphorylation after stimulation by LPS at 2 h post-stimulation and by IFNγ at 8 h post-stimulation in BV-2 cells. Results demonstrated that among the inhibitors tested, U0126 inhibited Ser505 phosphorylation in a dose-dependent manner whereas SB202190 and SP600125 were not effective (Fig. 3a, c). Similar results were observed with IFNγ (Fig. 3b, d). These results are consistent with the notion that phospho-ERK1/2 serves as the primary regulator of cPLA_2_ phosphorylation in microglia cells.

### LPS- and IFNγ-induced iNOS expression and NO production are significantly decreased in microglia deficient in cPLA_2_

**Fig. 4:**
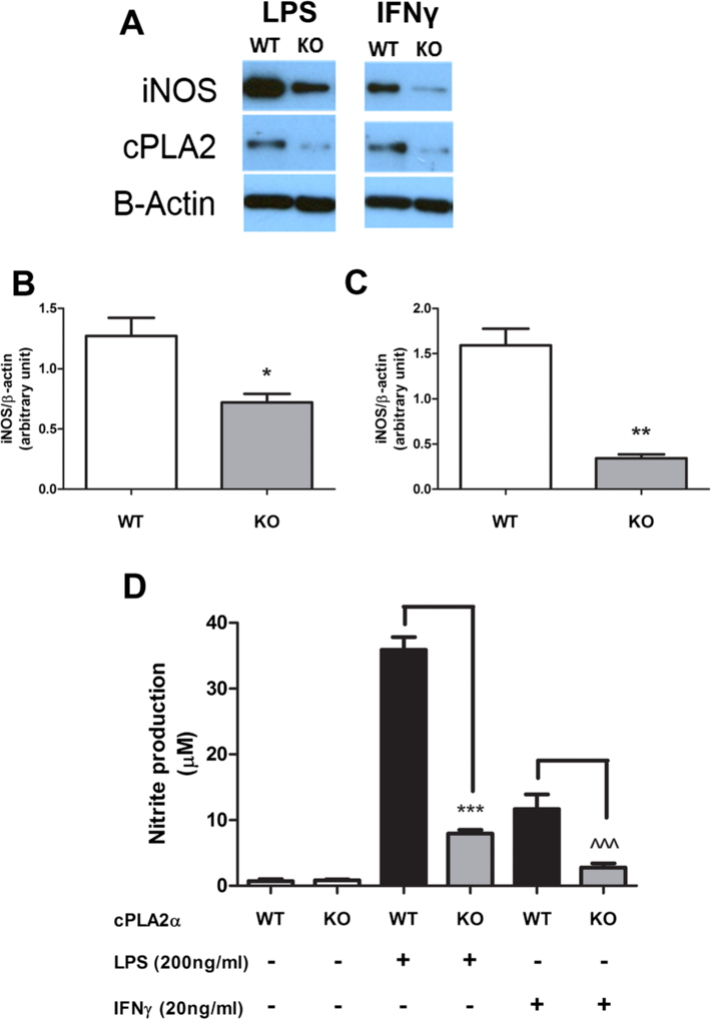
iNOS expression and NO production were significantly reduced in cPLA_2_ KO primary microglia culture compared with WT culture. DIV5–7 primary microglia culture isolated from cPLA_2_ KO or WT mice were stimulated with 200 ng/mL LPS or 20 ng/mL IFNγ for 24 h. Cells were then lysed, and proteins were collected/processed. **a** iNOS/cPLA_2_/β-actin expressions were demonstrated by Western blot, and **b**, **c** iNOS/ β-actin levels were quantified with the QuantityOne software. Results were expressed as the mean ± SEM (*n* = 3), and significant difference between the respective paired groups was determined by *t* test, *P < 0.05; **P < 0.01. **d** Conditioned mediums from 48 h post-stimulation samples were collected for determination of nitrite concentration with the Griess protocol. Results were expressed as the mean ± SEM (*n* = 3) and significant difference between the respective groups was determined by *t* test, ***P < 0.001; ^^^*P* < 0.001

In this study, primary microglial cells isolated from WT and cPLA_2_−/− homozygous KO mice brains were used to test for their ability to induce iNOS expression and NO production upon stimulation with LPS or IFNγ. As expected, cPLA_2_ expression was blunted in microglia isolated from the cPLA_2_ KO brain as compared to the WT brain (Fig. 4a). Under the same conditions, LPS-induced iNOS expression and NO production were significantly decreased in microglia isolated from the cPLA_2_ KO brain as compared to the WT brain (Fig. 4a, b, d). Similarly, IFNγ-induced iNOS expression and NO production were also significantly decreased in microglial cells from the cPLA_2_ KO brains (Fig. 4a, c, d). The concentration of LPS and IFNγ used in our experiment was shown not to cause significant cell death at the given time points (Additional file 1: Figure S1).

### LPS- and IFNγ-induced ROS from WT and cPLA_2_ KO microglia

**Fig. 5:**
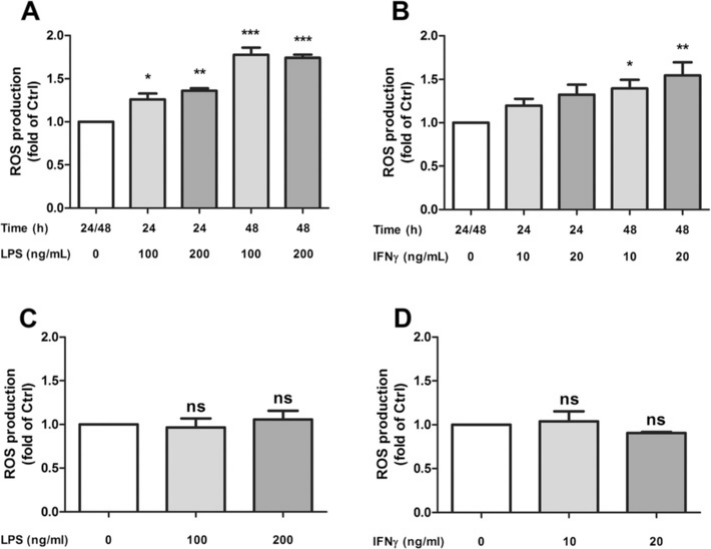
Primary microglia from cPLA_2_ KO mice did not show a significant increase in ROS production by LPS and IFNγ. Different concentrations of **a** LPS or **b** IFNγ, as well as incubation time were used to evaluate the ROS production by primary microglia isolated from wild-type mice. Based on the results from WT culture, ROS production was evaluated at 48 h post-stimulation with different doses of **c** LPS or **d** IFNγ in microglial cells from cPLA_2_ KO mice. ROS production was quantified by CM-H2DCFDA fluorescence as described in the text. Results were expressed as the mean ± SEM (*n* = 3 WT LPS, *n* = 8 WT IFNγ, *n* = 3 KO LPS, *n* = 3 KO IFNγ), and significant difference between the groups was determined by one-way ANOVA followed by Dunnett’s post-tests, *P < 0.05; **P < 0.01; ***P < 0.001

Our earlier study demonstrated the temporal profile and mechanism for LPS and IFNγ to induce ROS production in BV-2 microglial cells [39]. In this study, we attempted to compare ROS production between primary microglia isolated from WT and cPLA_2_ KO brains. In WT primary microglial cells, ROS production was maximally increased after stimulation with 200 ng/mL LPS and continued to increase for 48 h (Fig. 5a). ROS induced by IFNγ was not significantly increased at 24 h but continued to rise at 48 h (Fig. 5b). Under the same conditions and stimulus concentrations, neither LPS nor IFNγ managed to cause significant increase in ROS production in primary microglia isolated from cPLA_2_ KO brain (Fig. 5c, d).

### Pharmacological inhibition and siRNA of cPLA_2_ result in suppression of LPS- and IFNγ-induced NO production in BV-2 cells

**Fig. 6:**
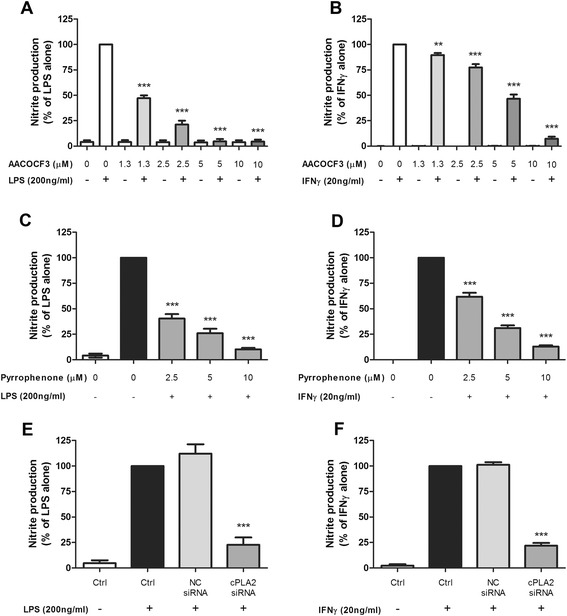
NO production in BV-2 cells after LPS or IFNγ stimulation was inhibited by cPLA_2_ pharmacological inhibitors or siRNA knockdown. BV-2 cells were starved for 4 h in serum-free DMEM. One hour prior to stimulation, cells were pretreated with the indicated concentrations of cPLA_2_ inhibitors: **a**, **b** AACOCF3 or **c**, **d** pyrrophenone. Cells were then stimulated with **a**, **c** 200 ng/mL LPS or **b**, **d** 20 ng/mL IFNγ. Alternatively, BV-2 cells were transfected with siRNA against cPLA_2_ for 24 h before being stimulated with **e** 200 ng/mL LPS or **f** 20 ng/mL IFNγ. For all experiments, conditioned mediums were collected 16 h post-stimulation and NO concentrations were measured by Griess protocol as described in the text. Results were expressed as the mean ± SEM (*n* = 3) and significant difference between the respective groups was determined by one-way ANOVA followed by Dunnett’s post-tests, **P < 0.01; ***P < 0.001

Based on the above data suggesting a link between cPLA_2_ and LPS/IFNγ-induced NO production, we further tested whether inhibition of cPLA_2_ by pharmacological inhibitors and by siRNA knockdown may alter the ability for LPS and IFNγ to stimulate NO in BV-2 microglial cells. In this study, two pharmacological inhibitors were used: AACOCF3, a non-specific PLA_2_ inhibitor known to suppress activity of both cPLA_2_ and iPLA_2_, and pyrrophenone, a specific cPLA_2_ inhibitor. As shown in Fig. 6a–d, both inhibitors showed dose-dependent suppression of NO generation 16 h after LPS and IFNγ stimulation. The doses of AACOCF3 and pyrrophenone used in this study were verified to not cause toxicity in the culture system using the WST-1 assay while aiming for reasonable level for maximum effect (data not shown).

RNA interference was further employed to ensure the inhibition observed above was not resulted from non-specific pharmacological effects. Using the cPLA_2_ siRNA (NM_008869, Qiagen) and RNAiMAX transfection reagent, we were able to knockdown cPLA_2_ by 70–80 %, based on protein expression by Western blot (Additional file 2: Figure S2). Under this condition, NO production was significantly suppressed in knockdown cultures as compared with control cultures (Fig. 6e, f).

### Pharmacological inhibition and siRNA of cPLA_2_ result in significant suppression of LPS- and IFNγ-induced ROS production in BV-2 cells

**Fig. 7:**
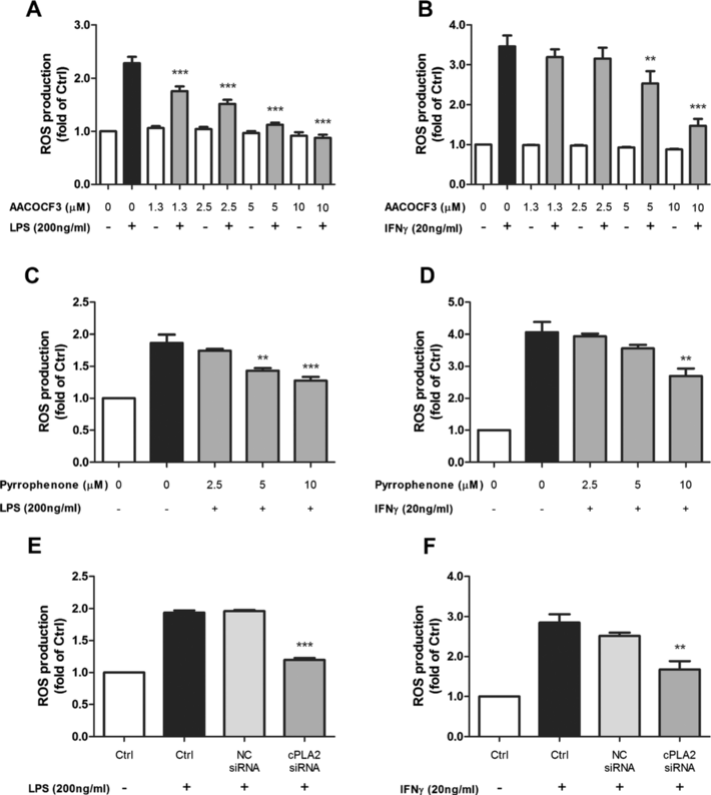
ROS production in BV-2 cells after LPS or IFNγ stimulation was inhibited by cPLA_2_ pharmacological inhibitors or siRNA knockdown. BV-2 cells were starved for 4 h in serum-free DMEM. One hour prior to stimulation, cells were pretreated with the indicated concentrations of cPLA_2_ inhibitors: **a**, **b** AACOCF3 or **c**, **d** pyrrophenone. Cells were then stimulated with **a**, **c** 200 ng/mL LPS or **b**, **d** 20 ng/mL IFNγ. Alternatively, BV-2 cells were transfected with siRNA against cPLA_2_ 24 h before being stimulated with **e** LPS or **f** IFNγ. For all experiments, ROS production was measured 12 h post-stimulation by CM-H2DCFDA fluorescence as described in the text. Results were expressed as the mean ± SEM (*n* = 3) and significant difference between the respective groups was determined by one-way ANOVA followed by Dunnett’s post-tests, **P < 0.01, ***P < 0.001

Our previous study demonstrated that ROS production from NADPH oxidase activation plays a major role in microglia activation and it precedes iNOS induction and NO production (Chuang et al. 2013). In this study, cPLA_2_ inhibitors and siRNA were used to test the link between cPLA_2_ and LPS- or IFNγ-induced ROS production in BV-2 cells. As shown in Fig. 7a–d, both AACOCF3 and pyrrophenone dose-dependently inhibited LPS- or IFNγ-induced ROS production where measured at 12 h post-stimulation. Similarly, siRNA also significantly diminished ROS induction by the two stimuli (Fig. 7e, f).

### cPLA_2_ inhibition prevents morphological changes associated with activation in primary microglia

**Fig. 8:**
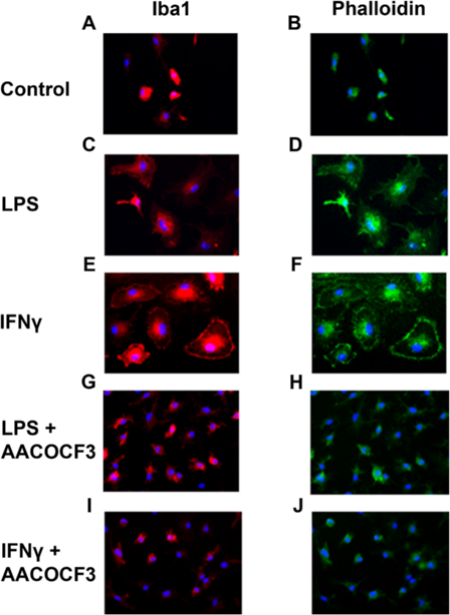
cPLA_2_ inhibition with AACOCF3 prevented morphological transformation of primary microglia associated with M1 activation. Immunocytochemical staining for Iba-1 and phalloidin was performed with DIV5 primary microglia culture with the following treatments for 24 h: **a**, **b** control; **c**, **d** 200 ng/mL LPS; **e**, **f** 20 ng/mL IFNγ; **g**, **h** 1 h of 5 μM AACOCF3 pretreatment with 200 ng/mL LPS for 24 h; **i**, **j** 1 h of 5 μM AACOCF3 pretreatment with 20 ng/mL IFNγ for 24 h. Iba-1 expression is represented by *red fluorescent signal*, phalloidin expression is represented by *green fluorescent signal*, and nuclear Hoechst staining is demonstrated in *blue*

In order to visualize the morphological changes of microglia under activation by LPS or IFNγ, primary microglia were cultured in coverslips, followed by fixation and immunostaining with Iba-1 (red), marker for microglial cells and phalloidin (green) for actin filaments. Undisturbed microglia cells were uniform in size and displayed small round cytoplasm with an off-centered nucleus, resembling the resting ramified phenotype (Fig. 8a, b). At 24 h after LPS stimulation, there was obvious expansion of cytoplasmic space with significant formation of filopodia (Fig. 8c, d). Cells treated with IFNγ appeared to show higher Iba1 staining with cytoplasm more spread out like a fried egg. Some also showed extensive budding around the periphery (Fig. 8e, f). There were also increased mitotic events as evident by cells with di-nuclei in both types of stimulation. Interestingly, microglia pretreated with 5 μM of AACOCF3 for 1 h prior to LPS or IFNγ stimulation showed preservation of morphology resembling closely to unstimulated ramified microglia in the control plates (Fig. 8g–j).

### Ca^2+^-independent PLA_2_ does not alter LPS- and IFNγ-induced iNOS/NO/ROS production

**Fig. 9:**
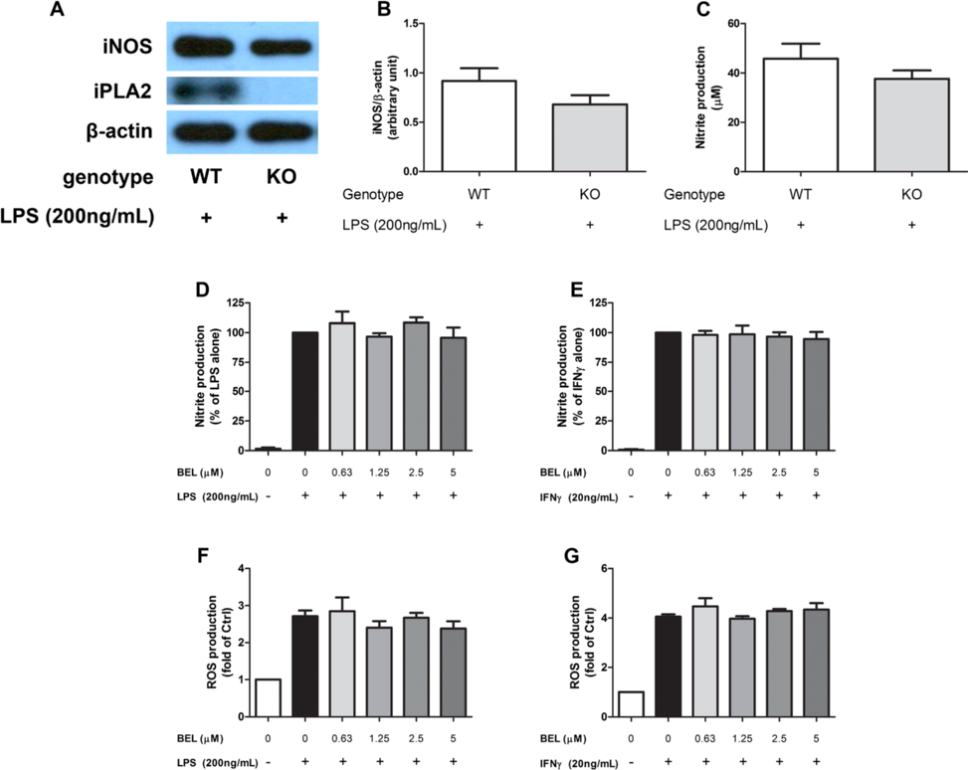
iPLA_2_ KO primary microglia culture did not alter LPS-induced NO/ROS production. DIV5–7 primary microglia culture isolated from iPLA_2_β KO or WT mice were stimulated with 200 ng/mL LPS for 24 h. Cells were then lysed and proteins were collected/processed. **a** iNOS/cPLA_2_/β-actin expressions were demonstrated by Western blot, and **b** iNOS/ β-actin levels were quantified with the QuantityOne software. **c** Conditioned mediums from 48 h post-stimulated samples were collected for determination of nitrite concentration with the Griess protocol. **d**–**g** BV-2 cells were serum starved for 3 h followed by incubation with indicated concentrations of BEL for 1 h before stimulated with **d**, **f** 200 ng/mL LPS or **e**, **g** IFNγ. **d**, **e** NO production was measured in conditioned medium 16 h post-stimulation by Griess protocol. **f**, **g** ROS production was measured 12 h post-stimulation by CM-H2DCFDA. Results were expressed as the mean ± SEM (*n* = 3) and significant difference between the groups was determined by *t* test (primary microglia) and one-way ANOVA followed by Dunnett’s post-tests (BV-2)

cPLA_2_ and iPLA_2_ are both constitutively expressed in most cell types, and are both possible contributors to AA production along with its downstream cascade. While we had established cPLA_2_ to play crucial role in microglia activation, it is also important to investigate whether iPLA_2_ may also play a role in this process. Using the Miltenyi Biotec MACS cell separation system, primary microglial cells were isolated from WT and iPLA_2_ KO brains. As shown in Fig. 9a, expression of iPLA_2_ was not observed in the iPLA_2_ KO brains, and stimulation with LPS did not result in a significant difference in iNOS expression and NO production between WT and KO microglia (Fig. 9b, c).

To further verify the results, we also tested whether selective iPLA_2_ inhibitor BEL (racemic) may have an effect on LPS- and IFNγ-induced NO and ROS production in BV-2 cells. As shown in Fig. 9d–g, results indicated that BEL did not significantly affect the amount of NO or ROS produced by either stimulus.

### cPLA_2_-dependent induction of NO or ROS in microglia does not go through COX-1/2

**Fig. 10:**
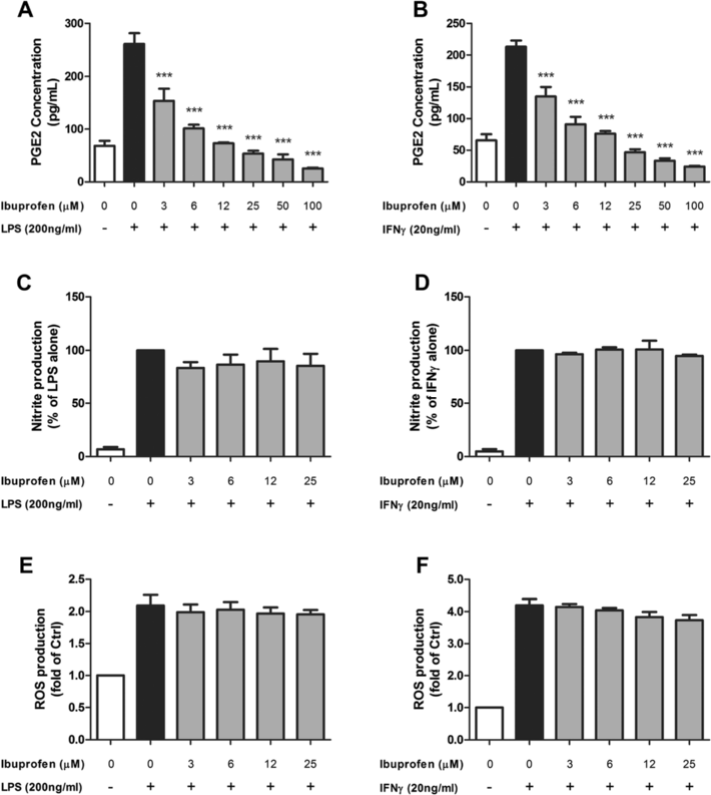
Ibuprofen dose-dependently inhibited the PGE2 production after LPS/IFNγ stimulation but did not affect the NO/ROS production in BV-2 cells. BV-2 cells were serum starved for 3 h followed by incubation with indicated concentrations of ibuprofen for 1 h before stimulated with **a**, **c**, **e** 200 ng/mL of LPS or **b**, **d**, **f** 20 ng/mL of IFNγ. **a**, **b** PGE2 production was measured in conditioned medium 16 h post-stimulation by the PGE2 ELISA assay as described in the text. **c**, **d** NO production was measured in conditioned medium 16 h post-stimulation by Griess protocol. **e**, **f** ROS production was measured 12 h post-stimulation by CM-H2DCFDA. Results were expressed as the mean ± SEM (*n* = 3) and significant difference between the respective groups was determined by one-way ANOVA followed by Dunnett’s post-tests, ***P < 0.001

cPLA_2_ is responsible for AA production and downstream eicosanoid production. AA can be converted by COX-1/2 into prostaglandin H2 which is further metabolized to prostaglandins, prostacyclin, and thromboxanes. Alternatively, AA can also go through the lipoxygenase (LOX) pathway to generate 5/12/15-hydroperoxyeicosatetraenoic acid (HPETE). COX-1/2 and prostaglandins have always been implicated in inflammatory processes and COX-1/2 remains a popular target of anti-inflammatory therapy by non-steroidal anti-inflammatory drugs (NSAIDs). In the following experiments, we tested the involvement of COX-1/2 in NO/ROS production in BV-2 cells. Using the ELISA protocol, we measured the concentration of PGE2 in conditioned medium of BV-2 microglial cell cultures after stimulation with LPS or IFNγ. We further investigated the effect of ibuprofen, a non-selective reversible COX-1/2 inhibitor, to inhibit PGE2 production. Results showed a dose-dependent inhibition of LPS- and IFNγ-induced PGE2 by ibuprofen (Fig. 10a, b). We further tested whether ibuprofen could inhibit LPS- and IFNγ-induced NO and ROS in BV-2 cells. Interestingly, ibuprofen did not exert inhibitory effects on either LPS- or IFNγ-induced NO and ROS production (Fig. 10c–f).

### LOX inhibition significantly suppresses cPLA_2_-dependent microglial induction of ROS and NO

**Fig. 11:**
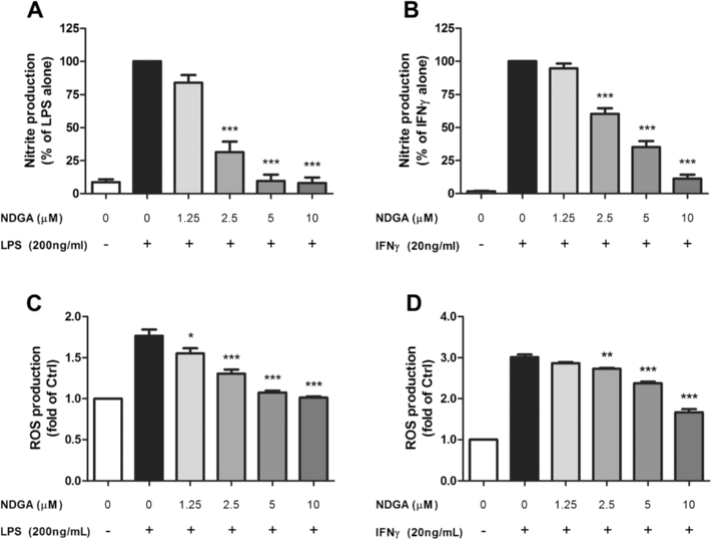
LOX inhibition mitigated NO or ROS production in BV-2 cells after LPS or IFNγ stimulation. BV-2 cells were serum starved for 3 h followed by incubation with indicated concentrations of NDGA for 1 h before stimulated with **a**, **c** 200 ng/mL LPS or **b**, **d** 20 ng/mL IFNγ. **a**, **b** NO production was measured in conditioned medium 16 h post-stimulation by Griess protocol. **c**, **d** ROS production was measured 12 h post-stimulation by CM-H2DCFDA. Results were expressed as the mean ± SEM (*n* = 3) and significant difference between the respective groups was determined by one-way ANOVA followed by Dunnett’s post-tests, *P < 0.05; **P < 0.01; ***P < 0.001

cPLA_2_-induced AA release can be metabolized by either COX or LOX. Since the above results indicated that COX played a minimal role in ROS/NO production, experiments were directed to test whether the LOX pathways may mediate LPS- and IFNγ-induced ROS and NO production. The LOX products have been shown to provide an important role in mediating the downstream inflammatory leukotrienes in neurodegenerative conditions and infectious processes [42]. When BV-2 cells were pretreated with NDGA, a non-selective LOX inhibitor, NO and ROS production was significantly suppressed in a dose-dependent manner (Fig. 11a–d).

**Fig. 12:**
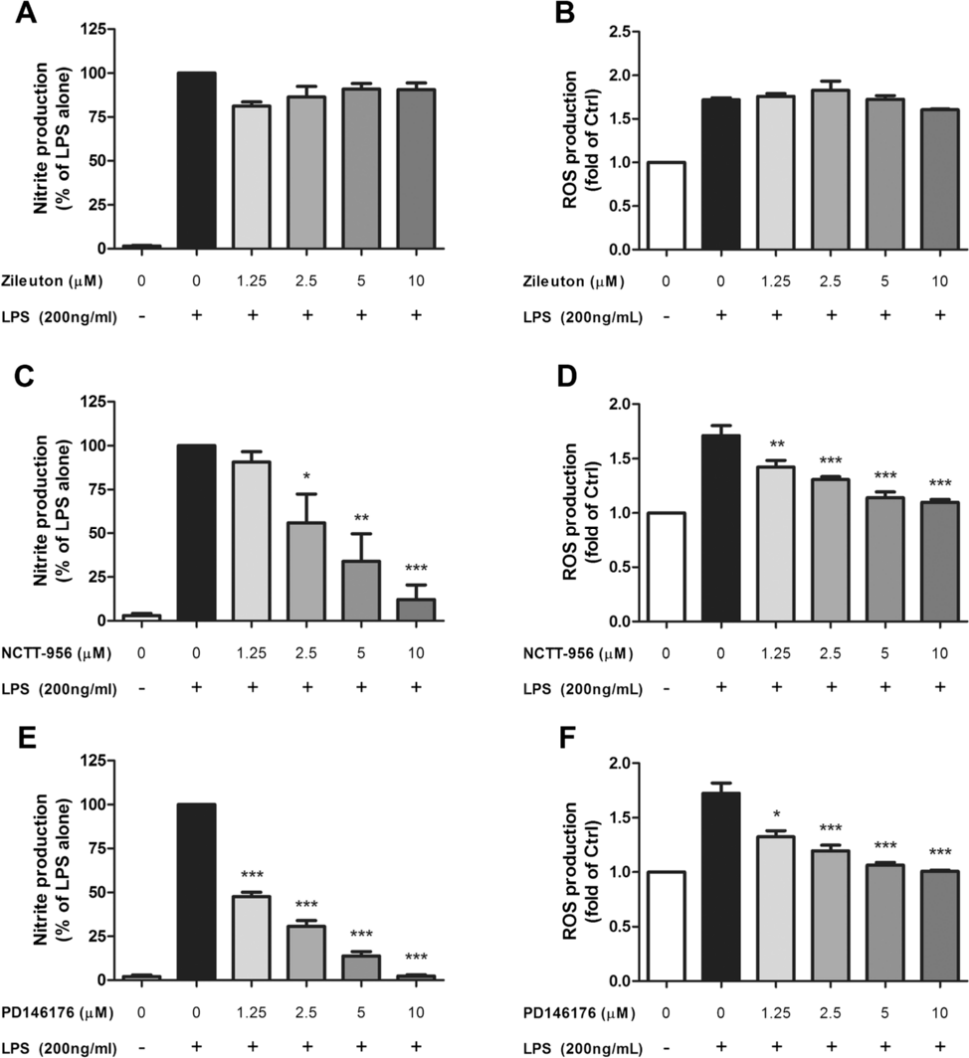
NO/ROS production by BV-2 cells after LPS stimulation was mitigated by LOX-12/15 inhibition, but not by LOX-5 inhibition. BV-2 cells were serum starved for 3 h followed by 1-h incubation with indicated concentrations of LOX inhibitors: **a**, **b** zileuton for LOX-5 inhibition; **c**, **d** NCTT-956 for LOX-12 inhibition; and **e**, **f** PD146176 for LOX-15 inhibition. The cells were then stimulated with 200 ng/mL LPS. **a, c, e** NO production was measured in conditioned medium 16 h post-stimulation by Griess protocol. **b**, **d**, **f** ROS production was measured 12 h post-stimulation with CM-H2DCFDA fluorescence. Results were expressed as the mean ± SEM (*n* = 3), and the significant difference between the respective groups was determined by one-way ANOVA followed by Dunnett’s post-tests, *P < 0.05; **P < 0.01; ***P < 0.001

Among the lipoxygenases, LOX-5, LOX-12, and LOX-15 are the most studied and are responsible for the generation of 5-HPETE, 12-HPETE, and 15-HPETE, respectively. To further investigate which LOX and its subsequent products were responsible for LPS- or IFNγ-induced ROS and NO production in microglia, we incorporated the use of zileuton, NCTT-956, and PD146176, previously described selective inhibitors for LOX-5, LOX-12, and LOX-15, respectively [43–45]. While zileuton at varying concentrations did not seem to affect production of either NO or ROS production by BV-2 cells after LPS stimulation (Fig. 12a, b), both NCTT-956 and PD146176 inhibited ROS/NO production in a concentration-dependent manner (Fig. 12c–f). Similar results were seen when BV-2 cells were stimulated by IFNγ (Additional file 3: Figure S3A–F). These results thus provided information that LPS- and IFNγ-induced ROS and NO in microglial cells may be regulated by LOX-12/15 and not LOX-5.

## Discussion

### cPLA_2_ plays a significant role in microglial activation

cPLA_2_ has been shown to play a significant role in mediating oxidative/nitrosative and inflammatory responses in neurons, astrocytes, and other cells [46, 47], but less attention has been paid to microglia. The findings of this study clearly demonstrate the involvement of cPLA_2_ in LPS- and IFNγ-induced ROS and NO production in microglial cultures. To our knowledge, this is the first study to use primary microglia prepared from cPLA_2_ and iPLA_2_ knockout mice to provide new evidence for the significant role of cPLA_2_ in microglial activation.

The comparison between cPLA_2_ knockout and wild-type primary microglia cells showed that cPLA_2_ not only plays a crucial role in activating the oxidative/inflammatory pathway, leading to generation and release of ROS/NO, but also to the overall morphological transformation of microglial cells after endotoxin/cytokine stimulation. To ensure that the findings are not a result of alternative mechanisms from long-term functional compensation in response to cPLA_2_ knockout, the same conclusion was reached using pharmacological inhibition, with AACOCF3 (non-specific) and pyrrophenone (specific), and RNA interference knockdown in BV-2 cells. Other studies using LPS but not IFNγ as a stimulator support this conclusion in rat primary microglia [30] and BV-2 cells [48]. On the other hand, while iPLA_2_ is also constitutively expressed in microglia and a target of AACOCF3, results here with iPLA_2_ KO and pharmacological inhibition with BEL showed that this PLA_2_ has little role in mediating ROS and NO production in the BV-2 microglial cells. This result is in slight contrast to the prior study by Strokin et al., who suggested that iPLA_2_ also contributes to the proinflammatory responses in LPS-treated astrocytes via Ca^2+^ signaling [49]. This difference may well be due to use of different cell type, i.e., microglia versus astrocytes.

### The ERK1/2-cPLA_2_-ROS-iNOS axis in microglial activation

cPLA_2_ is known to have multiple active serine residues susceptible for phosphorylation. Among these serine residues, Ser505 was identified to be phosphorylated by MAPK and served as an important regulator for cPLA_2_ activity and subsequent AA release [32, 41]. In the study by Pavicevic with vascular smooth muscle cells, phosphorylation of Ser515 by CaMKII was shown to precede Ser505 phosphorylation and phosphorylation of both Ser515 and 505 sites is required for activation of this enzyme [50]. In primary neurons in culture, stimulation with ionotropic glutamate receptor agonist such as NMDA resulted in ROS production through NADPH oxidase and rapid activation of ERK1/2 and cPLA_2_ [31]. In this study, we demonstrated that LPS and IFNγ each mediated a time-dependent increase in phospho-ERK1/2 and cPLA_2_ in both primary and BV-2 microglial cells. In both conditions, the time for increase in p-ERK1/2 preceded that for p-cPLA_2_. The relationship between p-ERK1/2 and cPLA_2_ was further confirmed by U0126, the MEK1/2-ERK1/2 inhibitor, which readily abrogated phosphorylation of cPLA_2_.

NADPH oxidase in microglia cells has been shown to play a significant role in neurodegenerative diseases, such as alcohol-induced neurodegeneration [51], Alzheimer’s disease [52], and Parkinson’s disease [53]. Previous study from our laboratory has demonstrated the production of ROS from NADPH oxidase to be upstream of NO production in BV-2 microglia cells. Our studies further demonstrated that in BV-2 microglial cells, LPS and IFNγ can individually stimulate ROS and iNOS/NO through phosphorylation of ERK1/2 [39, 40]. Study by Ribeiro et al. (2013) also demonstrated effects of cannabinoid receptor agonists and antagonists to suppress LPS-induced microglia activation via ERK1/2, cPLA_2_, and NF-κB. These results as well as ours placed LPS and IFNγ activation of ERK1/2 and cPLA_2_ upstream of the NF-κB transcriptional pathway. In rat microglial cells, Szaingurten-Solodkin observed a link between cPLA_2_ in NADPH oxidase and iNOS activated by aggregated Abeta1-42, a toxic peptide cleaved from the amyloid precursor protein [54]. In their study, it was proposed that cPLA_2_ regulated NADPH oxidase activity, which in turn caused upregulation of cPLA_2_, COX-1/2, and iNOS through an NF-κB-dependent mechanism. Taken together, our results with cPLA_2_ inhibitors as well as siRNA knockdown well demonstrated the role of cPLA_2_ in mediating ROS and NO production upon stimulation by LPS and IFNγ. Our results with primary microglia isolated from cPLA_2_ KO brain further validated the link between cPLA_2_ on ROS and NO production in these cells.

### Role of arachidonic acid and LOX in microglial activation

Earlier studies had linked cPLA_2_ or its downstream metabolites (i.e., AA or lysophospholipids) with ROS production from NADPH oxidase, although the exact mechanism remains to be investigated [55, 56]. In macrophages, there is evidence that cPLA_2_ can interact directly with NADPH oxidase subunits, namely p47phox and p67phox, which facilitate translocation of these subunits to membranes to form the active NADPH oxidase complex [55, 57]. Alternatively, downstream products of AA were proposed to be involved in ROS production from NADPH oxidase [58]. Activation of cPLA_2_ and subsequent release of AA has been shown in the production of an array of eicosanoids, including the production of prostaglandins and leukotrienes through activation of COX and LOX. However, the extent for this action is cell dependent [42]. A number of studies, including those from our own, have demonstrated the increase in PGE2 production upon stimulation with LPS and IFNγ in astroglial cells [59, 60]. In the present study with microglial cells, we showed that while the COX-1/2 inhibitor effectively inhibited LPS- and IFNγ-induced PGE2 production, this condition was not linked to the suppression of ROS and NO production by LPS and IFNγ.

While the action of COX-1/2 is well established in peripheral inflammation and a popular target for non-steroidal anti-inflammatory drugs (NSAIDs), its role in neuroinflammation is not well understood. Aspirin is used after acute stroke not for anti-inflammatory effect but rather for secondary prevention of atherosclerosis due to its antiplatelet properties through inhibition of prostaglandin and subsequent thromboxane A_2_ [61, 62]. Similarly, numerous recent large-scale double-blind placebo-controlled clinical trials have not found a beneficial effect of COX-1/2 inhibition in the treatment of neurological diseases where neuroinflammation is proposed to be involved, such as Alzheimer’s disease or depression [63, 64]. In agreement with our study, Minghetti and colleagues have also reported that microglial cell activation increased TNFα and COX-1/2, but this condition did not contribute to ROS/NO production [65, 66].

On the other hand, recent studies have generating growing recognition of LOX in mediating inflammation, and some have implicated its role in neuroinflammation. Lipoxygenases are known to mediate the pathophysiology of numerous inflammatory diseases, including asthma, immune disorders, and cancer. Parallel to the action of COX-1/2 for the biosynthesis of prostaglandins from AA, lipoxygenases mediate the biosynthesis of leukotrienes and eoxins from AA, all of which are eicosanoids that play a significant role in inflammation and immune function [42]. Of note, the ability for LOX-15 to generate eoxin has been identified as a novel pathway of inflammatory responses in mast cells and eosinophils [67] and as a promising novel target against asthma [68]. Genetic ablation of LOX-12/15, but not LOX-5, was shown to protect against denervation-induced muscle atrophy [69]. In the central nervous system, LOX-12/15 was shown to have increased expression in oligodendrocytes and microglia of periventricular leukomalacia [70], and disease phenotype was ameliorated by absence of LOX-12/15 in animal models of Alzheimer’s disease [71]. The LOX-5 pathway has also been associated with Alzheimer’s disease and other neurodegenerative conditions [72]. Although the mechanism of how LOX causes microglial activation and neuroinflammation remains to be elucidated, our results provided evidence suggesting that LOX activation in microglial cells plays a crucial role leading to induction of ROS and NO. To our knowledge, this is the first finding to biochemically connect cPLA_2_ pathway to oxidation and inflammatory responses in microglial cells through LOX. Future studies should further examine the specific involvement of LOX isoforms in proinflammatory gene expression and regulation of ROS production in microglial cells.

### cPLA_2_ as a therapeutic target against neurological diseases

Since the 1990s, cPLA_2_ has been demonstrated to be a favorable target for intervention against a wide range of neurological diseases. Using an experimental stroke model, Bonventre et al. was the first to show that cPLA_2_ knockout mice suffered less ischemic damage and had smaller infarct volume after transient middle cerebral artery occlusion [21]. Sanchez-Mejia et al. also demonstrated transgenic hAPP mice with cPLA_2_ knockout to exhibit significantly less cognitive deficit compared with cPLA_2_ intact transgenic hAPP mice, indicating a potential role of cPLA_2_ in the pathogenesis of Alzheimer’s disease [73]. AACOCF3, a non-selective cPLA_2_ and iPLA_2_ inhibitor, was discovered to be an effective pharmacological inhibitor of PLA_2_. Due to its physicochemical properties, it can readily penetrate into cell membranes. In thrombin-stimulated platelets, in Ca^2+^ ionophore-stimulated human monocytic cells, and in interleukin 1-stimulated mesangial cells, all liberation of AA is essentially blocked at a concentration of 5 to 20 μM [74, 75]. Since the discovery and popularization of AACOCF3, more recent studies further demonstrated the administration of cPLA_2_ pharmacologic inhibitor to offer protective effect against multiple neurological diseases. This includes the amelioration of focal ischemic damage in experimental stroke [28], prevention of secondary tissue damage in experimental autoimmune encephalitis, an in vivo model for multiple sclerosis [30], as well as preservation of neuronal survival and retention of motor function in a mouse model of spinal cord injury [29, 76].

While most of the studies suggested and focused on the action of cPLA_2_ in the neurons in the event of neurodegeneration and neuronal apoptosis, less attention was given to the potential role of cPLA_2_ in the microglia cells. While microglia activation plays an important role to limit neuronal damage and phagocytose cellular debris and foreign pathogens, M1 activation of microglia cells can also promote microglia-induced neuronal damage and further propagate ongoing neuroinflammation. The results of this study had shed light on one potential mechanism of M1 microglia activation, which can potentially be targeted and controlled. Interestingly, inhibition of cPLA_2_ by AACOCF3 was able to abrogate the morphological changes elicited by LPS and IFNγ in WT primary microglial cells. We believe that cPLA_2_ inhibition not only prevents microglia activation but more importantly becomes a viable therapeutic strategy to impede neuronal cell death by limiting secondary neuronal damage. In this regard, inhibiting PLA_2_ cascade has been considered an essential strategy for opposing microglia activation [77] and discovering new and synthetic inhibitors for PLA_2_ will be an important future endeavor for understanding and treatment of neurological disorders [78].

## Conclusions

This study demonstrated a crucial role of cPLA_2_ in the activation of microglial cells, specifically in LPS- and IFNγ-stimulated ROS and iNOS/NO production. In addition, cPLA_2_ also controls the morphological transformations associated with microglial activation. Upon looking at the downstream pathways, results show that LPS- and IFNγ-induced activation of ROS/NO is dependent on LOX-12/15 and not COX-1/2, thus offering new insights into the signaling pathway during microglia activation. Further studies are needed to better understand the molecular mechanisms underlying cPLA_2_ in microglial activation and how this may offer novel therapeutic options for the prevention and/or treatment of neuroinflammatory/neurodegenerative diseases.

## Additional files

Additional file 1: Figure S1. High concentrations of LPS and IFNγ were toxic to primary microglia at 24 h post-stimulation. Primary microglial cells were treated with various concentrations of either (A) LPS or (B) IFNγ. Twenty-four hours later, cell viability was measured with the WST-1 protocol as described in the text. Results were expressed as the mean ± SEM (*n* = 3) and significant difference compared with the control group was determined by one-way ANOVA followed by Dunnett’s post-tests, ***P* < 0.01, ****P* < 0.001. Additional file 2: Figure S2. cPLA_2_ protein expression level decreased significantly after siRNA knockdown. Representative blot demonstrating protein levels of cPLA_2_ and β-actin in BV-2 cells between groups: (1) control, (2) BV-2 cells were transfected with negative control siRNA for 24 h, and (3) BV-2 cells were transfected with siRNA against cPLA_2_ for 24 h. Additional file 3: Figure S3. NO/ROS production by BV-2 cells after IFNγ stimulation was mitigated by LOX-12/15 inhibition, but not by LOX-5 inhibition. BV-2 cells were serum starved for 3 h followed by 1-h incubation with indicated concentrations of LOX inhibitors: (A–B) zileuton for LOX-5 inhibition, (C–D) NCTT-956 for LOX-12 inhibition, and (E–F) PD146176 for LOX-15 inhibition. The cells were then stimulated with 20 ng/mL IFNγ. (A, C, E) NO production was measured in conditioned medium 16 h post-stimulation by Griess protocol. (B, D, F) ROS production was measured 12 h post-stimulation with CM-H2DCFDA fluorescence. Results were expressed as the mean ± SEM (*n* = 3) and significant difference between the respective groups was determined by one-way ANOVA followed by Dunnett’s post-tests, **P* < 0.05; ****P* < 0.001.
