# Supplementary figures and images for: Cytosolic phospholipase A2 plays a crucial role in ROS/NO signaling during microglial activation through the lipoxygenase pathway
Source: J Neuroinflammation. 2015 Oct 31;12:199. doi: 10.1186/s12974-015-0419-0 (PMC4628268; doi:10.1186/s12974-015-0419-0)

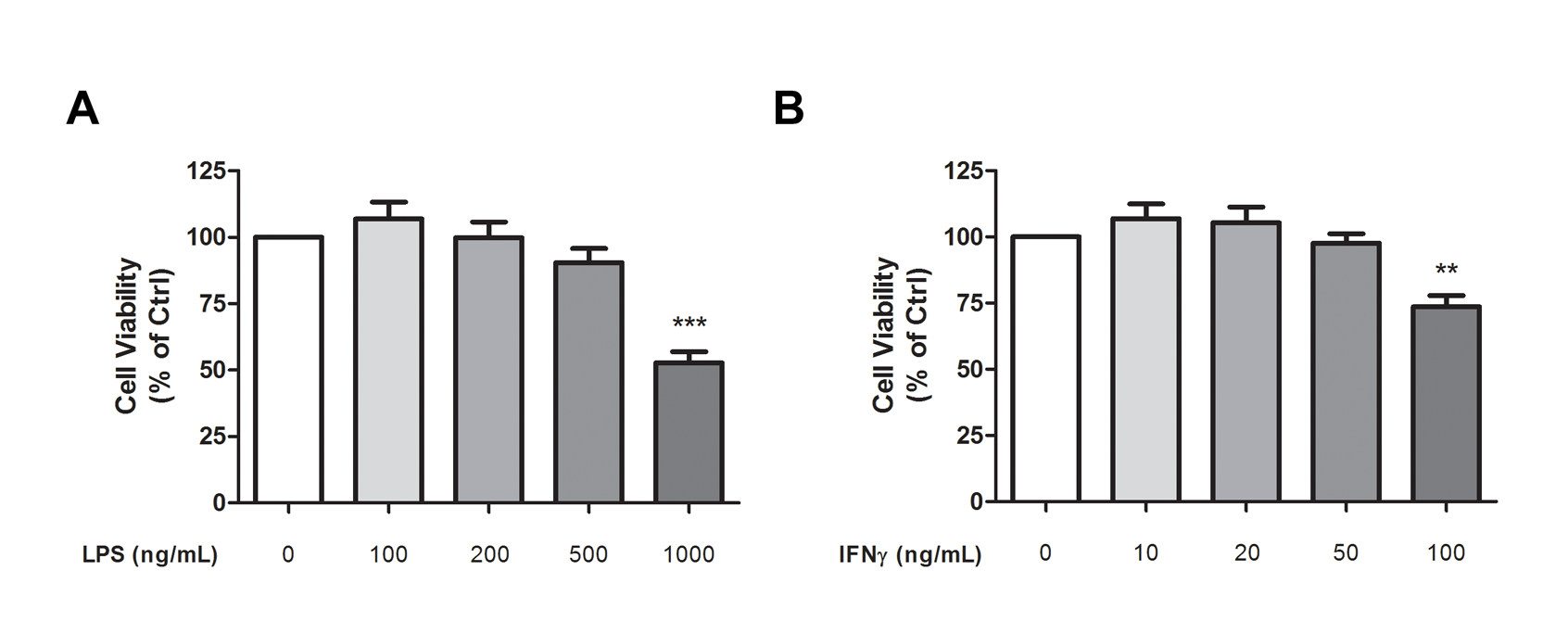

Supplement: Additional file 1: Figure S1. — High concentrations of LPS and IFNγ were toxic to primary microglia at 24 h post-stimulation. Primary microglial cells were treated with various concentrations of either (A) LPS or (B) IFNγ. Twenty-four hours later, cell viability was measured with the WST-1 protocol as described in the text. Results were expressed as the mean ± SEM (n = 3) and significant difference compared with the control group was determined by one-way ANOVA followed by Dunnett’s post-tests, **P < 0.01, ***P < 0.001. [file 12974_2015_419_MOESM1_ESM.tif]

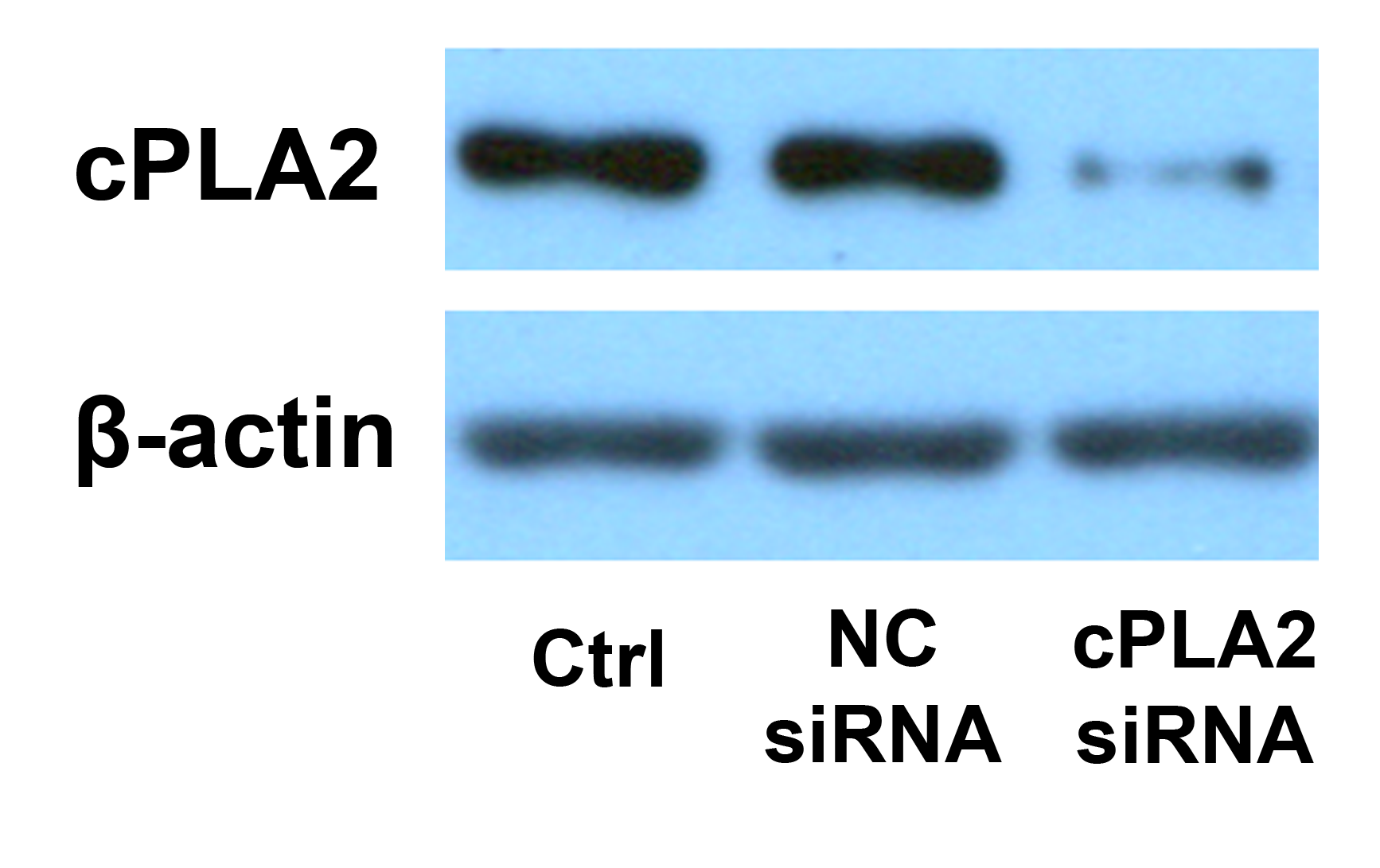

Supplement: Additional file 2: Figure S2. — cPLA2 protein expression level decreased significantly after siRNA knockdown. Representative blot demonstrating protein levels of cPLA2 and β-actin in BV-2 cells between groups: (1) control, (2) BV-2 cells were transfected with negative control siRNA for 24 h, and (3) BV-2 cells were transfected with siRNA against cPLA2 for 24 h. [file 12974_2015_419_MOESM2_ESM.tif]

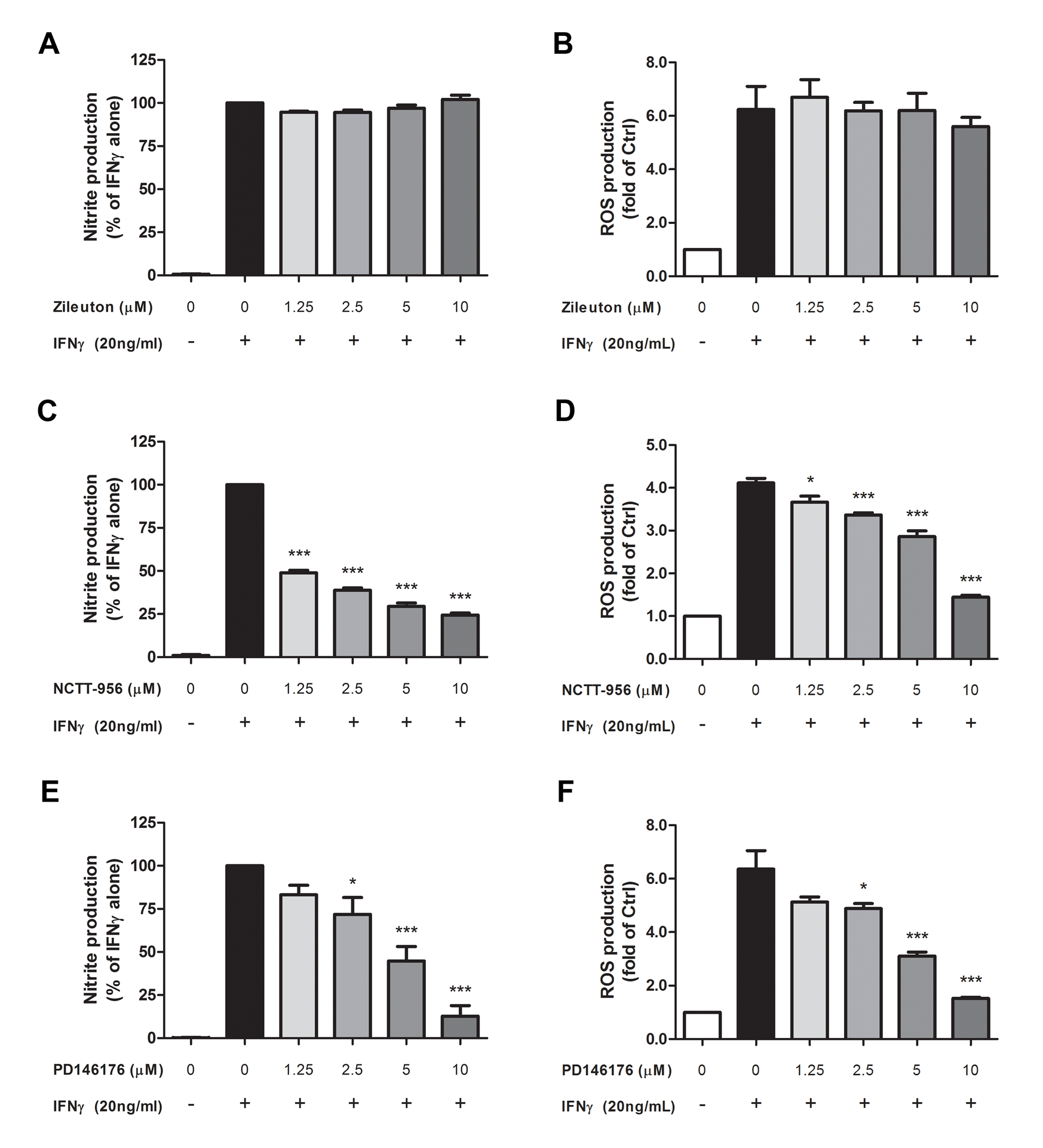

Supplement: Additional file 3: Figure S3. — NO/ROS production by BV-2 cells after IFNγ stimulation was mitigated by LOX-12/15 inhibition, but not by LOX-5 inhibition. BV-2 cells were serum starved for 3 h followed by 1-h incubation with indicated concentrations of LOX inhibitors: (A–B) zileuton for LOX-5 inhibition, (C–D) NCTT-956 for LOX-12 inhibition, and (E–F) PD146176 for LOX-15 inhibition. The cells were then stimulated with 20 ng/mL IFNγ. (A, C, E) NO production was measured in conditioned medium 16 h post-stimulation by Griess protocol. (B, D, F) ROS production was measured 12 h post-stimulation with CM-H2DCFDA fluorescence. Results were expressed as the mean ± SEM (n = 3) and significant difference between the respective groups was determined by one-way ANOVA followed by Dunnett’s post-tests, *P < 0.05; ***P < 0.001. [file 12974_2015_419_MOESM3_ESM.tif]
